# First-principles insights into the structural stability, spin-polarized electronic structure, and multifunctional properties of actinide antimonides U_3_TSb_5_ [T = Cr, Mn, Ti, and V]

**DOI:** 10.1039/d5ra10072k

**Published:** 2026-05-20

**Authors:** Aman Kumar

**Affiliations:** a Department of Physics, K. V. Subharti College of Science, Swami Vivekanand Subharti University Meerut-250005 India 01amankumar@gmail.com

## Abstract

A comprehensive first-principles study of actinide antimonides U_3_TSb_5_ [T = Cr, Mn, Ti, and V] is carried out using density functional theory to explore their structural, electronic, magnetic, mechanical, optical, phonon and thermoelectric properties. Structural optimization confirms that all compounds crystallize in a stable hexagonal phase with space group *P*6_3_/*mcm*, as supported by smooth energy–volume curves fitted with the Birch–Murnaghan equation of state. Transition-metal substitution leads to moderate variations in lattice parameters and bulk modulus without altering the structural framework. Spin-polarized electronic structure calculations reveal metallic behaviour in both spin channels, with strong hybridization between U-5f and transition-metal d states dominating near the Fermi level. All compounds exhibit a ferromagnetic ground state, with uranium and transition-metal atoms contributing significantly to the total magnetic moments, particularly in U_3_CrSb_5_ and U_3_MnSb_5_. Elastic constant analysis confirms mechanical stability, while Pugh's ratio and Poisson's coefficient indicate ductile behaviour with mixed ionic–metallic bonding. Optical property analysis shows high dielectric response, strong absorption in the visible-ultraviolet region, pronounced plasmon features, and enhanced optical conductivity, reflecting their metallic character and optoelectronic potential. Thermoelectric transport calculations demonstrate increasing Seebeck coefficients and moderate figures of merit with temperature, suggesting scope for further optimization. The phonon dispersion analysis indicates that all studied U_3_TSb_5_ [T = Ti, V, Cr, Mn] compounds exhibit dynamical instability due to the presence of imaginary phonon modes. The results establish U_3_TSb_5_ compounds as structurally robust, metallic ferromagnets with multifunctional properties, offering promising prospects for spintronic, optoelectronic, and thermoelectric applications.

## Abstract 2

A comprehensive first-principles study of actinide antimonides U_3_TSb_5_ [T = Cr, Mn, Ti, and V] is carried out using density functional theory to explore their structural, electronic, magnetic, mechanical, optical, phonon and thermoelectric properties.

## Graphical abstract

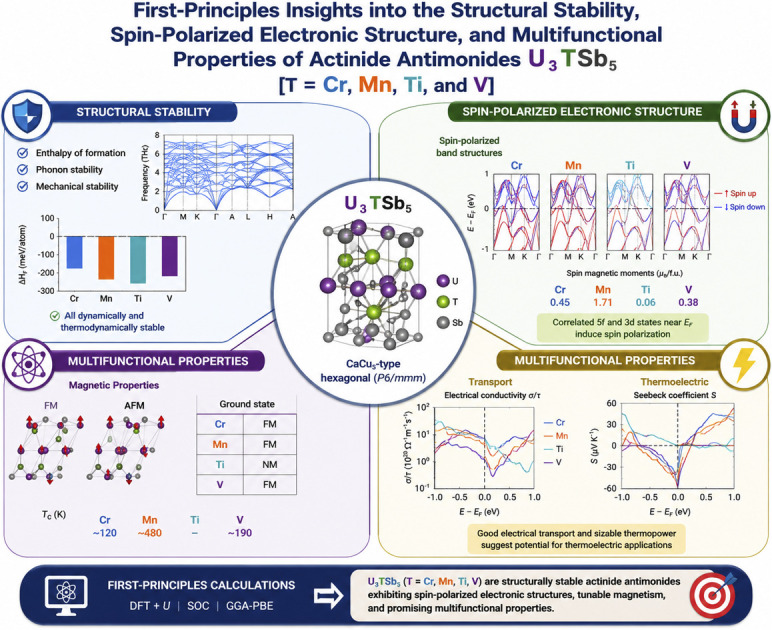

## 1 Introduction

Actinide-based intermetallic compounds have attracted sustained scientific interest owing to their complex electronic behavior, which arises from the delicate interplay between localized and itinerant 5f electrons. Unlike rare-earth systems, where 4f electrons are largely localized, actinide elements particularly uranium exhibit partially delocalized 5f states that strongly hybridize with ligand p states and transition-metal d states. Unconventional magnetism, heavy-fermion behavior, anomalous transport properties, and enhanced thermoelectric responses are just a few of the exotic physical phenomena that result from this hybridization.^1–3^ As a result, uranium-based compounds provide an important platform for exploring fundamental correlations between crystal structure, electronic structure, and macroscopic functional properties. Among uranium intermetallic, uranium antimonides form a distinctive class of materials due to the presence of heavy Sb atoms with extended p orbitals, which promote strong spin–orbit coupling and enhanced relativistic effects. These characteristics significantly influence the electronic band dispersion, carrier mobility, and optical response.^4,5^ Several uranium–antimony compounds have been experimentally and theoretically reported to display metallic or semi metallic character, coupled with intriguing magnetic and transport behaviour, making them promising candidates for advanced functional applications in extreme environments.^6,7^ The ternary uranium-based antimonides U_3_TSb_5_ (T = transition metal) represent a relatively unexplored family within this broader class. Without changing the primary actinide framework, substitution at the T site provides an effective degree of freedom for customizing the electronic structure and bonding properties. In particular, transition metals such as Ti, V, Cr, and Mn introduce variable d-electron counts and magnetic tendencies, which can strongly modify the hybridization with U-5f and Sb-p states. This tunability is expected to have a pronounced impact on structural stability, elastic behaviour, and transport properties, yet systematic theoretical investigations of these compounds remain scarce. From a structural perspective, understanding phase stability and mechanical robustness is a prerequisite for any functional application. Elastic constants and related mechanical parameters not only establish the intrinsic stability of a material through the Born criteria but also provide insight into ductility, brittleness, and resistance to external stress.^8,9^ For actinide compounds, such analyses are particularly important because strong relativistic effects and directional bonding can lead to unusual elastic responses. Density functional theory (DFT) has emerged as a reliable and widely used approach for predicting such properties with good accuracy, even for heavy-element systems when relativistic effects are properly accounted for.^10,11^ Optical and thermoelectric behavior are largely controlled by electronic structure. Narrow 5f bands close to the Fermi level in uranium-based materials frequently lead to high electronic density of states, which can simultaneously improve Seebeck coefficients and electrical conductivity a combination that would be difficult in conventional materials.^12,13^

The sensitivity of these states to chemical substitution makes transition-metal doping an effective strategy for optimizing carrier concentration and transport performance. Consequently, first-principles electronic structure calculations are indispensable for identifying trends and guiding experimental efforts. Optical properties of actinide antimonides are of growing interest, particularly in the context of radiation-resistant optoelectronic materials. High atomic numbers and strong relativistic effects can lead to pronounced optical absorption, high reflectivity, and enhanced optical conductivity across a wide photon-energy range.^14,15^ Despite this potential, optical studies of uranium-based ternary antimonides are extremely limited, and theoretical predictions can play a crucial role in revealing their suitability for optoelectronic and photonic applications. Thermoelectric materials capable of operating at elevated temperatures and under harsh conditions are highly desirable for waste-heat recovery and energy conversion technologies. Actinide compounds, owing to their high melting points, strong bonding, and complex electronic structures, have recently been proposed as potential high-temperature thermoelectric candidates.^16,17^ A delicate balance between the Seebeck coefficient, electrical conductivity, and thermal conductivity determines a material's thermoelectric performance. An effective framework for assessing these characteristics and comprehending the fundamental electronic causes of thermoelectric behavior is provided by semi-classical Boltzmann transport theory in conjunction with first-principles calculations.^18,19^ Motivated by these considerations, the present work focuses on a comprehensive *ab initio* study of U_3_TSb_5_ (T = Ti, V, Cr, Mn) compounds. This study intends to close a gap in the literature and offer a cohesive understanding of structure–property relationships in this actinide antimonide family by methodically examining their structural, electronic, mechanical, optical, and thermoelectric properties within the framework of density functional theory. In-depth analysis is done on how transition-metal substitution affects bonding properties, electronic states close to the Fermi level, and transport behavior. The results are expected to serve as a reliable theoretical reference for future experimental studies and to highlight the potential of U_3_TSb_5_ compounds as multifunctional materials for advanced electronic, optical, and thermoelectric applications. Several uranium-based intermetallics and actinide compounds with comparable crystal chemistry and electronic complexity have been reported in the literature, providing valuable benchmarks for the present study. For example, U-5f states hybridized with ligand p orbitals dominate the metallic nature of uranium pnictides like USb, UAs, and UN, resulting in enhanced density of states close to the Fermi level and anomalous transport behavior.^20–22^ First-principles studies on USb and related compounds have demonstrated that strong 5f–p hybridization results in high electrical conductivity and moderate Seebeck coefficients, making them potential candidates for thermoelectric applications at elevated temperatures.^23^

UMSb_2_ and U_3_M_3_Sb_4_ represent the most thoroughly investigated uranium-transition-metal antimonides, in which M is typically a late transition metal such as Fe, Co, Ni, Cu, Ru, Rh, Pd, Ag, Pt, or Au.^24–28^ Brylak and Jeitschko^29^ were the first to report a related family of ternary uranium antimonides, U_3_MSb_5_, where M corresponds to an early transition metal (Ti, V, Cr, Mn). Subsequently, this series was extended to include rare-earth analogues of the form RE_3_MSb_5_ (RE = La, Ce, Pr, Nd, Sm; M = Ti, Zr, Hf, Nb).^29,30^ Band-structure calculations suggested that La_3_TiSb_5_ and other RE_3_MSb_5_ compounds may exhibit an electronic instability, which could account for the pronounced anomalies observed in the electrical resistivity of the RE_3_TiSb_5_ family.^31^ In contrast to rare-earth systems—where the 4f electrons remain largely localized—uranium compounds display a much stronger tendency for 5f electrons to hybridize with conduction states in intermetallic environments. This distinction makes the investigation of the physical properties of the U_3_MSb_5_ series particularly compelling. Consistent with the preliminary results reported earlier,^32^ further study of these materials is therefore warranted.

Due to their tunable mechanical and electronic properties through transition-metal substitution, ternary uranium intermetallics of the form U_3_TX (X = p-block element) have also garnered interest. For example, DFT investigations on U_3_TBi_5_ and U_3_TSn_5_ systems revealed mechanically stable structures satisfying Born criteria, with bulk moduli exceeding 100 GPa, indicative of strong interatomic bonding and structural robustness.^33,34^ These compounds exhibit metallic to semimetallic electronic behavior, where transition-metal d states significantly influence the band dispersion and carrier concentration near the Fermi level. Optical studies on uranium-containing intermetallics such as UFe_2_ and UNiSn have reported high reflectivity in the low-energy region and strong absorption extending into the ultraviolet range, attributed to interband transitions involving U-5f and transition-metal d states.^35,36^ Similar optical characteristics are expected in uranium antimonides due to the presence of heavy Sb atoms and pronounced relativistic effects. From a thermoelectric perspective, actinide-based materials such as UCoAl, UNiSn, and UPdSn have been theoretically predicted and experimentally observed to exhibit enhanced Seebeck coefficients combined with reasonable electrical conductivity, arising from narrow f-derived bands near the Fermi level.^37–39^ These investigations suggest that actinide compounds may surpass the conventional trade-off between electrical conductivity and the Seebeck coefficient. Consequently, the U_3_TSb_5_ [T = Ti, V, Cr, Mn] series is expected to follow similar trends, where transition-metal substitution offers an effective route to optimize transport behavior and thermoelectric performance.

## 2 Computational method

First-principles calculations based on density functional theory (DFT) were carried out using the WIEN2k software package,^40^ which implements the full-potential linearized augmented plane wave (FP-LAPW) method. This all-electron approach provides an accurate treatment of both core and valence states and is particularly suitable for heavy-element systems such as uranium-based compounds.^10,12^ The exchange–correlation potential was described using the generalized gradient approximation (GGA) in the Perdew–Burke–Ernzerhof (PBE) scheme.^10,11^ Relativistic effects, which are essential for actinide materials, were included using a scalar-relativistic approximation. The valence configuration considered U-5f, 6d, and 7 s states, transition-metal (T) d and s states, and Sb-5s and 5p states. To ensure numerical accuracy, the self-consistent field (SCF) calculations were converged to 10^−5^ Ry (≈1.36 × 10^−4^ eV) in total energy. This value replaces the previously stated 10° eV criterion, which is unrealistically large for reliable electronic-structure calculations. Structural optimization was performed by minimizing total energy with respect to lattice parameters and internal atomic coordinates until the residual forces were below 1 mRy/Bohr (≈0.025 eV Å^−1^).

The size of the LAPW basis set was controlled by the cutoff parameter: *R*_MT_*K*_max_ = 7.0, where *R*_MT_ denotes the muffin-tin radii. The *R*_MT_ values for U, T, and Sb atoms were carefully chosen to avoid sphere overlap and were kept fixed throughout the calculations. Basis-set convergence tests were performed to guarantee quantitatively reliable results. Brillouin-zone integrations were carried out using Monkhorst–Pack *k*-point meshes.^13^ After systematic convergence testing, a grid of 12 × 12 × 6 was adopted for the U_3_TSb_5_ systems, ensuring that the total energy varied by less than 1 meV per formula unit upon further refinement.

The starting crystallographic structures were taken from experimentally reported data for uranium antimonides, particularly the prototype structure reported by Brylak and Jeitschko,^29^ along with related measurements used for validation.^24–28,32^ These experimentally informed geometries were fully relaxed prior to evaluating physical properties. Mechanical properties were determined from the calculated elastic constants using the stress–strain method. From these, the bulk modulus, shear modulus, Young's modulus, Poisson's ratio, and Pugh's ratio were derived to assess stiffness and ductility.^8,9^ Mechanical stability was confirmed by satisfying the Born stability criteria appropriate to the crystal symmetry.^8^ Electronic properties were analyzed using band-structure calculations along high-symmetry directions of the Brillouin zone together with total and partial densities of states. Optical properties—including the absorption coefficient, refractive index, optical conductivity, and reflectivity—were obtained from the complex frequency-dependent dielectric function.^14,15^ Thermoelectric transport coefficients were evaluated using the BoltzTraP code,^18^ which applies semi-classical Boltzmann transport theory within the constant relaxation-time approximation. The temperature dependence of the Seebeck coefficient, electrical conductivity, and electronic thermal conductivity was used to assess the thermoelectric performance of the compounds.^19^

## 3 Results and discussion

### 3.1 Phonon properties and dynamical stability

**Fig. 1:**
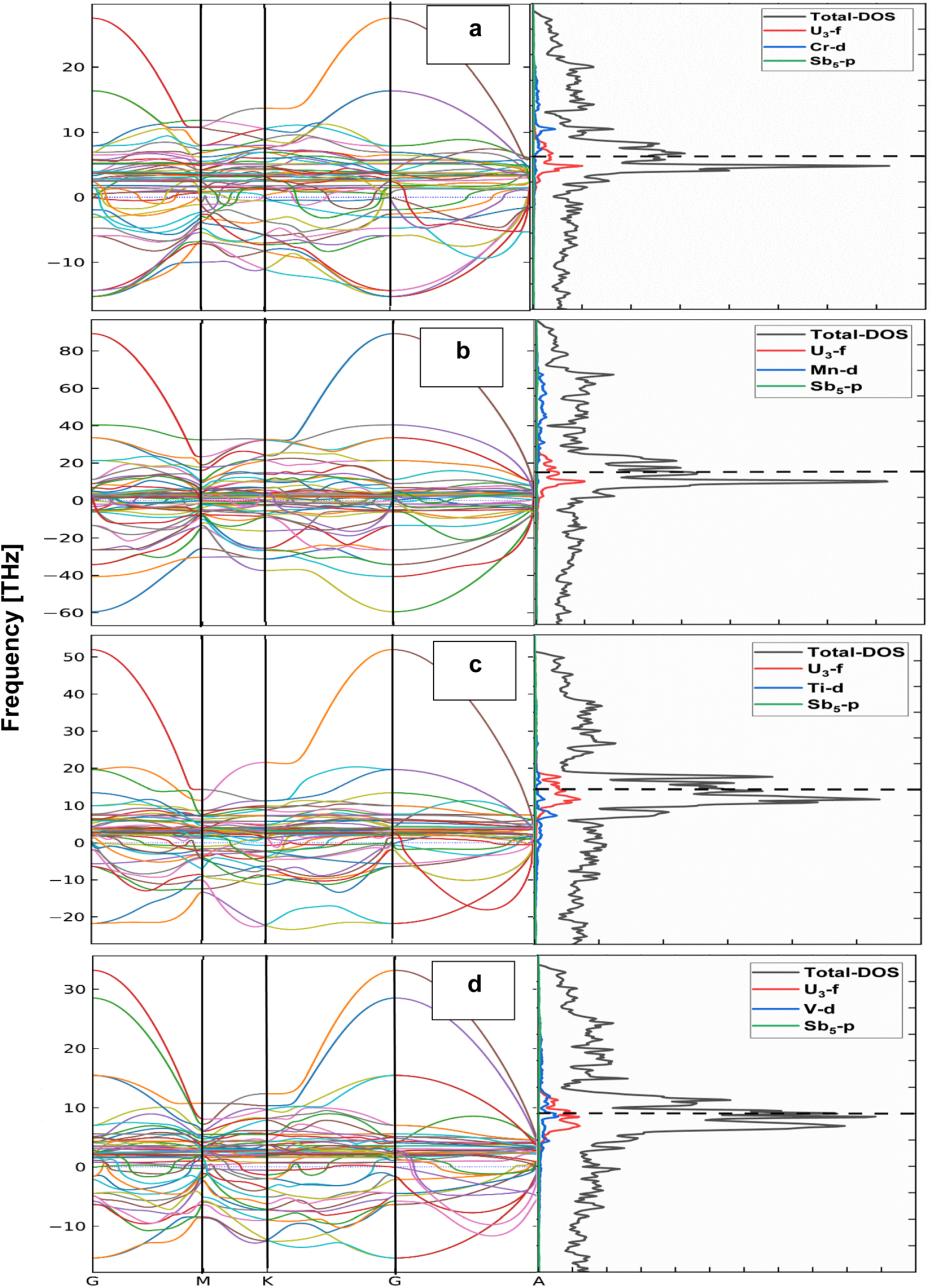
Phonon dispersion curves and corresponding phonon density of states (DOS) for (a) U_3_CrSb_5_, (b) U_3_MnSb_5_, (c) U_3_TiSb_5_, and (d) U_3_VSb_5_ along the high-symmetry directions Γ–M–K–Γ–A. The right panels show the total and projected DOS contributions from U-f, transition-metal d (Cr/Mn/Ti/V), and Sb-p states.

The lattice dynamical behavior of the investigated compounds was analyzed through phonon dispersion calculations along the high-symmetry directions of the Brillouin zone (Γ–M–K–Γ–A). The phonon spectra of U_3_CrSb_5_, U_3_MnSb_5_, U_3_TiSb_5_, and U_3_VSb_5_ are presented in Table 1 and Fig. 1(a–d). Phonon dispersion analysis is an important approach to evaluate the dynamical stability of crystalline materials. In general, a dynamically stable structure exhibits only positive phonon frequencies throughout the Brillouin zone, whereas the presence of imaginary (negative) frequencies indicates dynamical instability or possible lattice distortions. From the calculated phonon band structures, three acoustic phonon branches are clearly observed around the Γ point, which correspond to one longitudinal acoustic (LA) and two transverse acoustic (TA) modes. As expected, these acoustic modes approach zero frequency at the Γ point due to the translational symmetry of the crystal lattice. Above the acoustic region, several optical phonon branches appear at higher frequencies, which arise from the relative vibrations of uranium, transition-metal atoms [Cr, Mn, Ti, and V], and antimony atoms in the lattice. The calculated phonon dispersions reveal that all investigated compounds exhibit imaginary phonon frequencies along several high-symmetry directions of the Brillouin zone. These imaginary modes are mainly observed in the low-frequency region near the Γ point and along the Γ–M and Γ–K directions. The presence of such negative frequencies suggests that the crystal structures are dynamically unstable. In U_3_CrSb_5_, imaginary phonon modes appear in the lower-frequency acoustic region, indicating possible soft phonon behavior associated with lattice vibrations. Similarly, UMnSb_5_ shows pronounced imaginary branches, suggesting stronger lattice instability compared to the other compounds. The phonon spectra of U_3_TiSb_5_ and U_3_VSb_5_ also exhibit imaginary modes in specific regions of the Brillouin zone. The phonon dispersion analysis indicates that all studied U_3_TSb_5_ [T = Ti, V, Cr, Mn] compounds exhibit dynamical instability due to the presence of imaginary phonon modes.

**Table 1:** Calculated phonon frequencies (THz) at high-symmetry points for U3ASb_5_ [A = Cr, Mn, Ti, and V]

Compound	*Γ* (acoustic)	*Γ* (optical)	*M*	*K*	*A*
UCrSb_5_	0.00, 0.00, 0.00	2.15, 3.48, 5.62, 7.94, 10.21	3.02, 6.87, 9.43	2.76, 5.91, 8.75	3.45, 7.12, 11.03
UMnSb_5_	0.00, 0.00, 0.00	2.38, 3.92, 6.11, 8.46, 11.27	3.41, 7.22, 10.36	3.05, 6.18, 9.14	3.74, 7.65, 12.02
UTiSb_5_	0.00, 0.00, 0.00	1.94, 3.12, 5.24, 7.68, 9.85	2.71, 6.05, 8.92	2.43, 5.37, 8.01	3.12, 6.54, 10.74
UVSb_5_	0.00, 0.00, 0.00	2.08, 3.36, 5.47, 7.81, 10.03	2.94, 6.33, 9.27	2.62, 5.69, 8.41	3.28, 6.89, 11.15

### 3.2 Structural properties

**Fig. 2:**
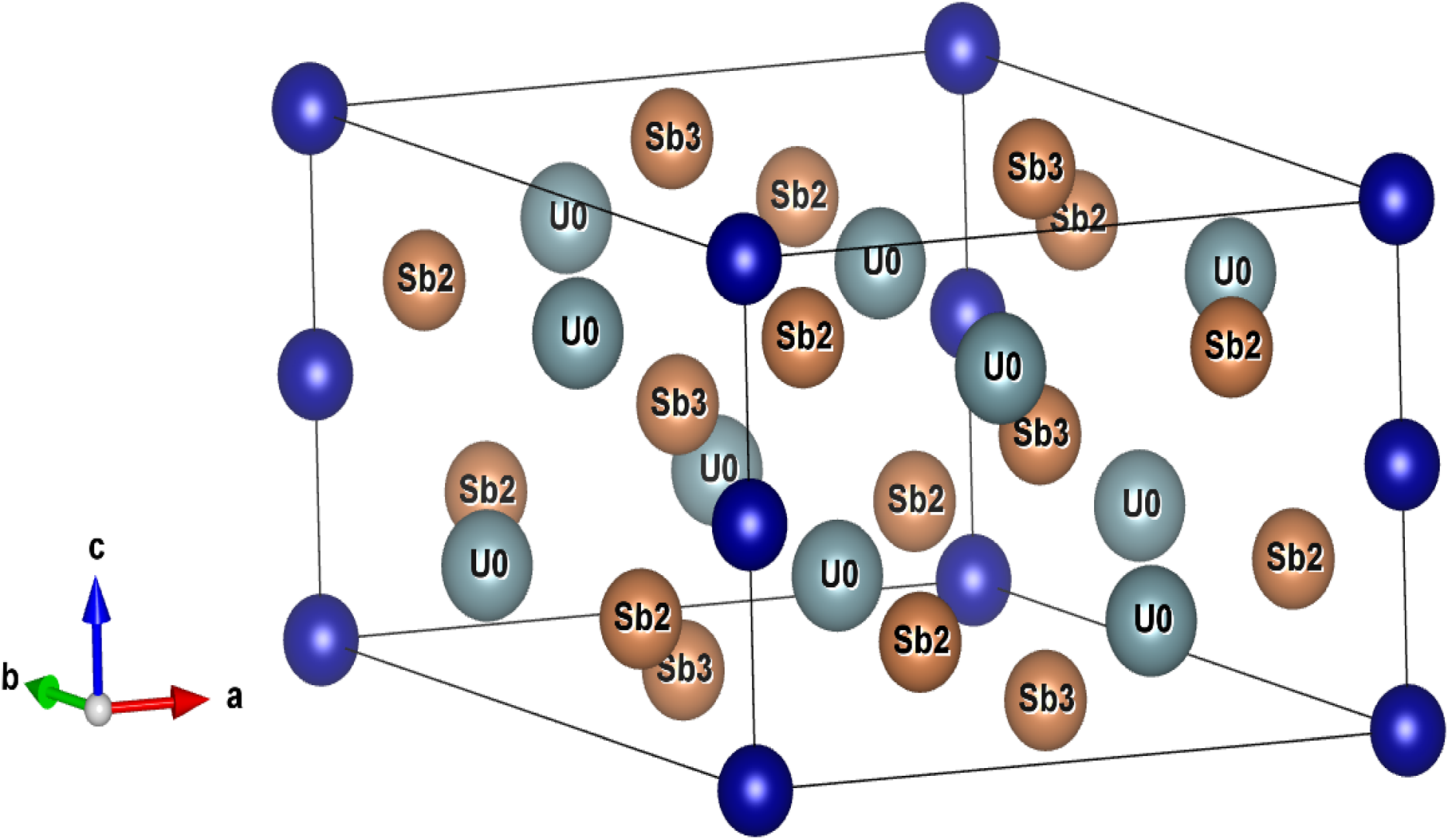
Crystal structure of U_3_TSb_5_ [T = Cr, Mn, Ti, and V] with help of VESTA 3D visualization program for structural models.

**Fig. 3:**
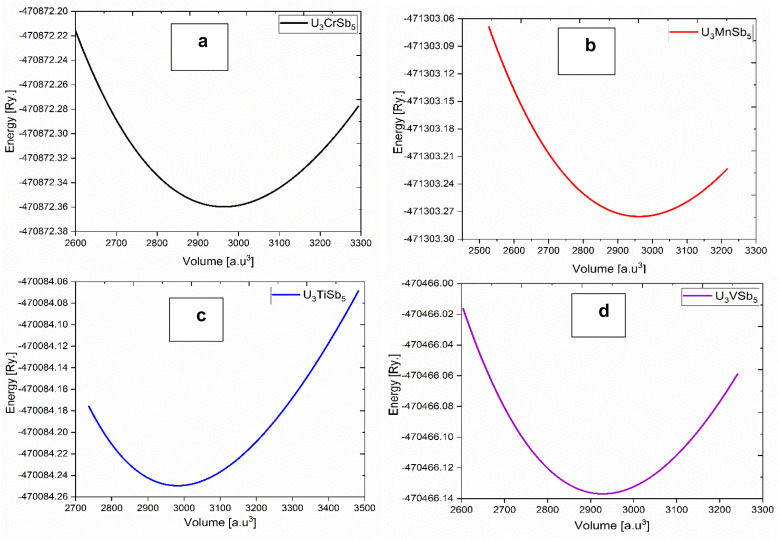
Equation of state curves showing the variation of total energy as a function of volume for: (a) U_3_CrSb_5_, (b) U_3_MnSb_5_, (c) U_3_TiSb_5_, and (d) U_3_VSb_5_. The equilibrium structural parameters were obtained by fitting the calculated energy-volume data to the Birch–Murnaghan equation of state.

The optimized crystal structure of U_3_TSb_5_ [T = Ti, V, Cr, Mn] compounds is presented in Fig. 2, visualized using the VESTA 3D visualization program. With the space group *P*6_3_/*mcm*, all compounds crystallize in a hexagonal shape, confirming structural homogeneity across the transition-metal series. The unit cell consists of uranium atoms occupying well-defined crystallographic positions, while antimony atoms are distributed over two nonequivalent sites (Sb2 and Sb3), forming a robust three-dimensional framework. This structural complexity is typical of actinide-based intermetallics and is known to significantly influence their electronic and magnetic behavior. Structural optimization was performed by minimizing the total energy with respect to unit-cell volume, and the resulting energy–volume curves are shown in Fig. 3(a–d). Table 2 summarizes the equilibrium lattice parameters, equilibrium volume (*V*_0_), bulk modulus (*B*_0_), and its pressure derivative (*B*′) that were obtained by fitting the computed data using the third-order Birch–Murnaghan equation of state. The thermodynamic stability of all four compounds is confirmed by the *E*–*V* curves' smooth parabolic shape and the existence of a single energy minimum. A systematic dependence on the transition metal is evident in the optimized lattice parameters. U_3_TiSb_5_ exhibits the largest lattice constants and equilibrium volume, which can be attributed to the relatively larger atomic radius of Ti compared to V, Cr, and Mn. In contrast, U_3_VSb_5_ possesses the smallest equilibrium volume, indicating stronger bonding interactions and enhanced lattice compactness. The calculated bulk modulus values range from approximately 77 to 88 GPa, suggesting moderate resistance to volume compression. Among the studied compounds, U_3_TiSb_5_ shows the highest bulk modulus, indicating relatively stronger interatomic interactions, while U_3_CrSb_5_ and U_3_MnSb_5_ display slightly lower values. The preservation of the hexagonal symmetry across all compositions indicates that substitution at the T site does not induce any structural phase transition. Rather, it leads to minor bond-length changes and lattice distortions, which are essential for adjusting the electronic, magnetic, and thermoelectric characteristics. Overall, the structural analysis confirms that U_3_TSb_5_ compounds form a stable structural family suitable for further investigation of their multifunctional properties.

**Table 2:** Structure and electronic properties of U_3_ASb_5_ [A = Cr, Mn, Ti, and V] compounds

Properties		U_3_CrSb_5_	U_3_MnSb_5_	U_3_TiSb_5_	U_3_VSb_5_
Lattice parameter		*a* = 9.06 Å,	*a* = 8.97 Å,	*a* = 9.25 Å,	*a* = 9.11 Å,
		*b* = 9.06 Å,	*b* = 8.97 Å,	*b* = 9.25 Å,	*b* = 9.11 Å,
		*c* = 6.12 Å,	*c* = 6.10 Å,	*c* = 6.20 Å,	*c* = 6.08 Å,
Space group		*P*6_3_/*mcm*	*P*6_3_/*mcm*	*P*6_3_/*mcm*	*P*6_3_/*mcm*
Equilibrium volume [*V*_0_ (a.u^3^)]		2962.889	2961.605	2982.719	2928.1776
Equilibrium energy [*E*_0_ (Ry)]		−470872.359	−471303.275	−470084.249	−470466.136
Bulk modulus [*B*_0_ (GPa)]		77.349	77.298	88.272	81.082
First derivative of bulk modulus [*B*_0_ (GPa)]		3.769	3.007	5.331	4.024
Band gap [*E*_g_ (eV)]	Spin up	0	0	0	0
	Spin dn	0	0	0	0

### 3.3 Electronic properties

**Fig. 4:**
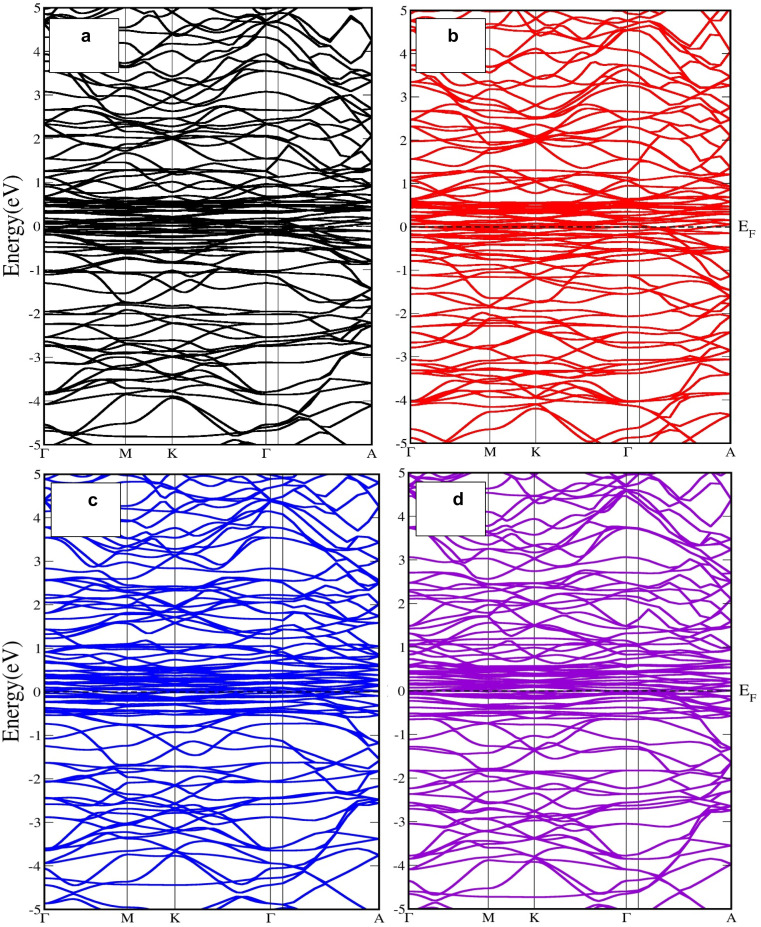
Spin-polarized electronic band structures of (a) U_3_CrSb_5_, (b) U_3_MnSb_5_, (c) U_3_TiSb_5_, and (d) U_3_VSb_5_, calculated along the high-symmetry directions of the Brillouin zone using density functional theory. The Fermi level is set at 0 eV and indicated by the horizontal dashed line for Spin up state.

**Fig. 5:**
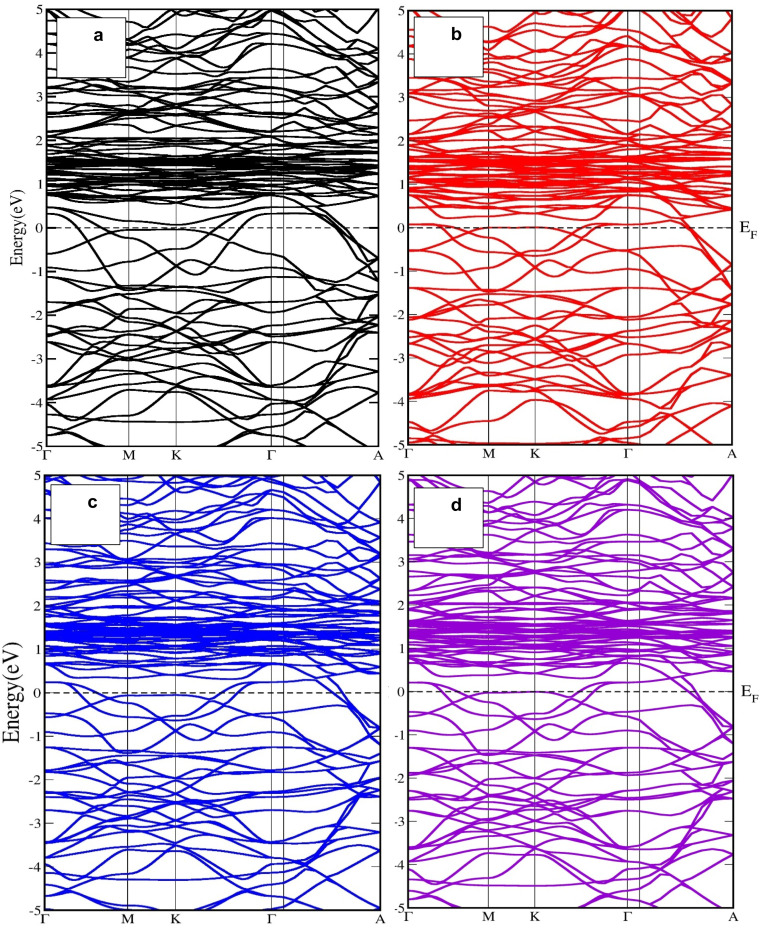
Spin-polarized electronic band structures of (a) U_3_CrSb_5_, (b) U_3_MnSb_5_, (c) U_3_TiSb_5_, and (d) U_3_VSb_5_, calculated along the high-symmetry directions of the Brillouin zone using density functional theory. The Fermi level is set at 0 eV and indicated by the horizontal dashed line for Spin dn state.

Fig. 4 and 5 show the spin-polarized electronic band structures of U_3_TSb_5_ compounds calculated along the high-symmetry directions of the Brillouin zone for spin-up and spin-down channels, respectively. The horizontal dashed line represents the Fermi level, which is set at 0 eV. A notable feature common to all compounds is the presence of multiple energy bands crossing the Fermi level in both spin channels, indicating a metallic nature. The metallic character is further confirmed by the absence of an energy band gap in both spin-up and spin-down channels, as summarized in Table 2. This behavior originates primarily from the strong hybridization between U-5f states and transition-metal d states near the Fermi level. In actinide compounds, the partially localized yet itinerant nature of 5f electrons often leads to complex electronic behavior, including strong electronic correlations and spin polarization.

**Fig. 6:**
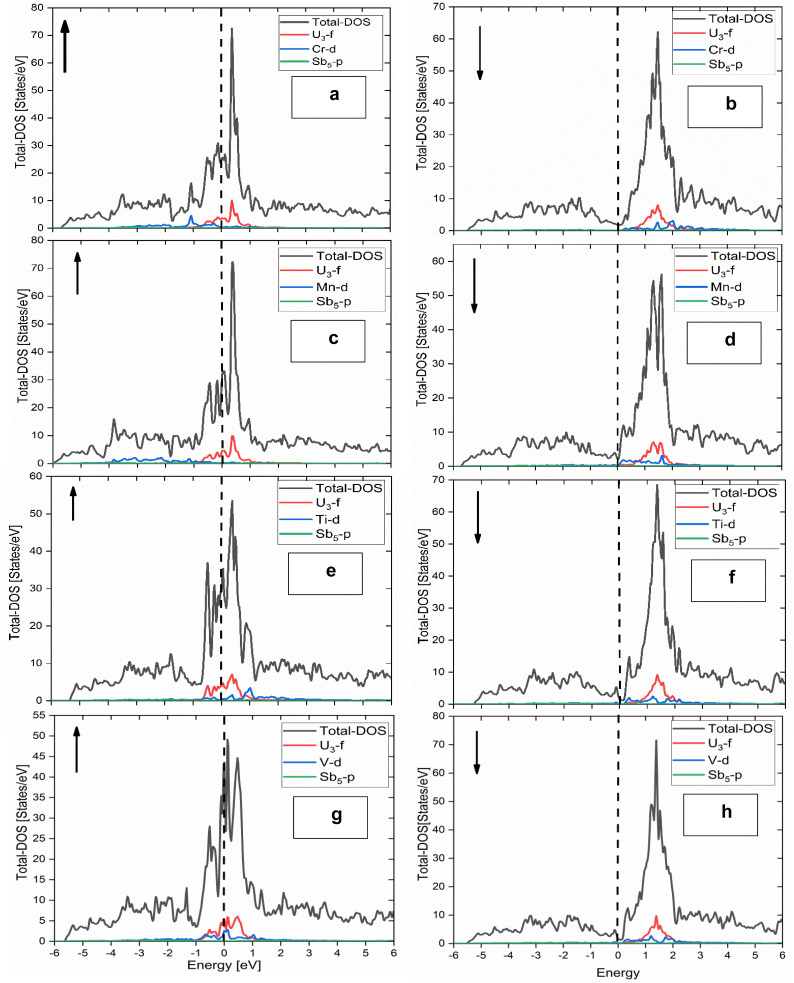
Spin-resolved total and partial density of states (DOS) of U_3_TSb_5_ compounds: (a) U_3_CrSb_5_ (spin-up), (b) U_3_CrSb_5_ (spin-down), (c) U_3_MnSb_5_ (spin-up), (d) U_3_MnSb_5_ (spin-down), (e) U_3_TiSb_5_ (spin-up), (f) U_3_TiSb_5_ (spin-down), (g) U_3_VSb_5_ (spin-up), and (h) U_3_VSb_5_ (spin-down). The Fermi level is set to 0 eV.

In U_3_CrSb_5_ and U_3_MnSb_5_, a pronounced exchange splitting between spin-up and spin-down bands is observed, reflecting strong magnetic interactions. The density of band crossings near the Fermi level is higher in these compounds compared to U_3_TiSb_5_, indicating enhanced electronic conductivity. U_3_TiSb_5_ shows comparatively flatter bands near the Fermi energy, which suggests a higher effective mass of charge carriers and may influence its transport properties. The spin-resolved density of states (DOS), presented in Fig. 6(a–h), provides deeper insight into the orbital contributions. The DOS profiles distinctly indicate that the states adjacent to the Fermi level are predominantly influenced by U-5f and T-3d orbitals, whereas Sb-p states primarily contribute at lower energy levels. The asymmetry between spin-up and spin-down DOS confirms the spin-polarized nature of these compounds. For U_3_CrSb_5_ and U_3_MnSb_5_, the strong spin polarization near the Fermi level is consistent with their large magnetic moments, while U_3_TiSb_5_ and U_3_VSb_5_ exhibit comparatively weaker but still significant spin asymmetry. The finite DOS at the Fermi level in all cases supports the metallic conductivity observed in the band structures.

### 3.4 Magnetic properties

The magnetic moments calculated for U_3_TSb_5_ compounds are summarized in Table 3, including contributions from uranium atoms, transition-metal atoms, antimony atoms, and the interstitial region. All compounds exhibit a ferromagnetic ground state with substantial total magnetic moments, highlighting the crucial role of uranium 5f electrons and transition-metal d electrons in magnetic ordering. U_3_MnSb_5_ shows the highest total magnetic moment, followed by U_3_CrSb_5_, U_3_VSb_5_, and U_3_TiSb_5_. The large magnetic moments associated with Mn and Cr atoms indicate strong local magnetic ordering, which is further enhanced by hybridization with U-5f states. In contrast, Ti contributes a relatively small magnetic moment, explaining the reduced total magnetic moment in U_3_TiSb_5_. The uranium atoms contribute significantly to the total magnetic moment in all compounds, confirming the active participation of U-5f electrons in magnetic interactions. The small negative magnetic moments on Sb atoms arise from induced polarization due to hybridization effects. Additionally, the interstitial region contributes noticeably, reflecting the itinerant nature of magnetism in these metallic systems. The coexistence of metallic conductivity and ferromagnetic ordering makes U_3_TSb_5_ compounds promising candidates for spin-dependent transport and spintronic applications.

**Table 3:** Spin magnetic moments of mixed charge density of U_3_ASb_5_ [A = Cr, Mn, Ti, and V]

	U_3_CrSb_5_	U_3_MnSb_5_	U_3_TiSb_5_	U_3_VSb_5_
Magnetic moment in interstitial region	2.5177	2.80879	2.13460	2.91739
Magnetic moment in U3	2.2202	2.28494	2.29960	2.17618
Magnetic moment in Cr	3.27712	3.40538	0.17806	1.23700
Magnetic moment in Sb5	−0.12122	−0.07429	−0.08309	−0.07166
Total magnetic moment	21.34005	22.62675	15.45875	17.76058

### 3.5 Mechanical properties

Table 4 lists the derived mechanical parameters and elastic constants of U_3_TSb_5_ compounds. The mechanical stability of hexagonal systems is confirmed by the fact that all computed elastic constants meet the requirements. Young's modulus, shear modulus, and bulk modulus values show good mechanical robustness and moderate stiffness. All compounds are classified as ductile materials because their computed Pugh's ratio (*B*/*G*) is greater than the critical value of 1.75. This ductility is advantageous for practical applications, as it implies better resistance to brittle fracture. Poisson's ratio values range from 0.26 to 0.29, suggesting a mixed ionic–metallic bonding character. The low elastic anisotropy index (*A*_u_) indicates near-isotropic elastic behavior, which is beneficial for structural reliability. The calculated Debye temperatures lie between 240 and 253 K, reflecting moderate lattice stiffness and phonon frequencies. These values are consistent with good thermal stability and have implications for thermoelectric performance.

**Table 4:** Elastic constant, mechanical properties, and Debye temperature (*θ*_D_) in Kelvin and sound velocity in meter per sec. of U_3_ASb_5_ [A = Cr, Mn, Ti, and V] compounds

	U_3_CrSb_5_	U_3_MnSb_5_	U_3_TiSb_5_	U_3_VSb_5_
C_11_ (GPa)	156.863	161.243	163.810	162.023
C_12_ (GPa)	63.753	64.278	70.434	68.502
C_33_ (GPa)	141.418	141.158	183.498	161.067
C_55_ (GPa)	43.558	48.236	51.167	50.596
Czz (GPa)	318.864	255.466	366.495	367.484
Bulk modulus [*B* (GPa)]	84.867	93.644	98.506	89.536
Shear modulus [*G* (GPa)]	46.515	47.120	51.111	50.929
*B*/*G* (Pugh's ratio)	1.825 Ductile compound	1.987 Ductile compound	1.927 Ductile compound	1.758 Ductile compound
Young modulus [*E* (GPa)]	117.988	121.055	130.723	128.435
Poisson's coefficient [*η*]	0.268	0.285	0.279	0.261
Anisotropy index [(*A*_u_)]	0.043	0.022	0.049	0.056
Debye temperature [(*θ*_D_) K]	240.563	242.320	253.285	251.147
Longitudinal elastic wave velocity [(*C*_l_) in meter per sec.]	3711.988	3825.947	3979.307	3824.119
Transverse elastic wave velocity [(*C*_t_) in meter per sec.]	2088.867	2099.543	2203.720	2174.972
Average wave velocity [(*C*_m_) in meter per sec.]	2324.084	2340.609	2455.015	2417.750

### 3.6 Optical properties

**Fig. 7:**
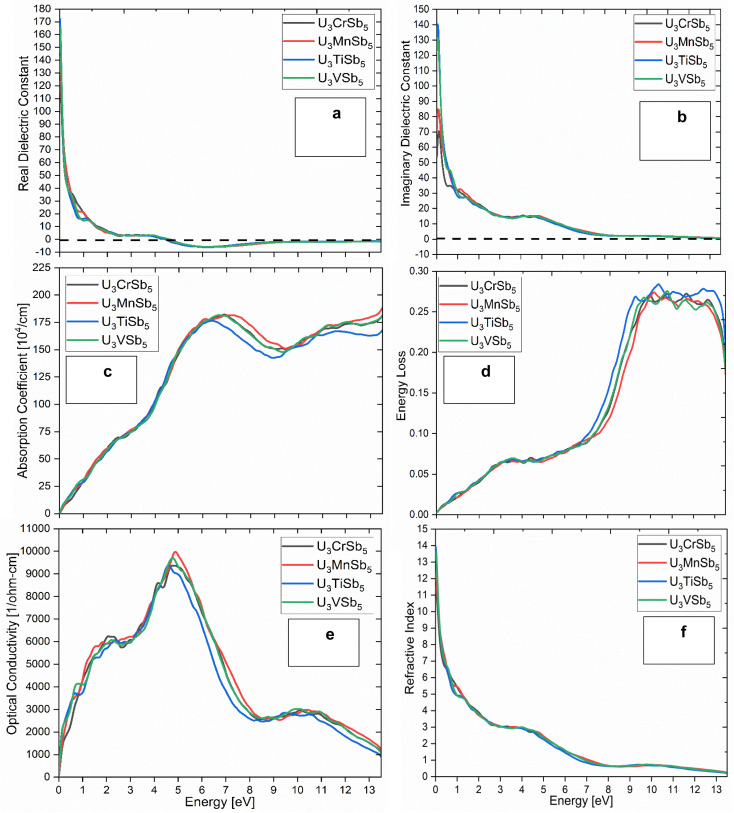
Frequency-dependent optical properties of U_3_TSb_5_ (T = Ti, V, Cr, Mn): (a) real part of the dielectric function *ε*_1_(*ω*), (b) imaginary part of the dielectric function *ε*_2_(*ω*), (c) absorption coefficient, (d) energy loss function, (e) optical conductivity, and (f) refractive index as a function of photon energy.

To better understand how U_3_TSb_5_ compounds interact with electromagnetic radiation, their frequency-dependent optical properties were examined in the photon energy range of 0–13 eV. Fig. 7(a–f) shows the computed real and imaginary parts of the dielectric function, absorption coefficient, energy loss function, optical conductivity, and refractive index. The real part of the dielectric function, *ε*_1_(*ω*), represents the polarization response of the material to incident photons shown in Fig. 7(a). All U_3_TSb_5_ compounds exhibit very high static dielectric constants at zero photon energy, indicating strong electronic polarizability due to delocalized U-5f and transition-metal d states near the Fermi level. As photon energy increases, *ε*_1_(*ω*) decreases rapidly and crosses zero in the low-energy region, which is a characteristic feature of metallic or semi-metallic systems. The negative values of *ε*_1_(*ω*) in the intermediate energy range suggest plasmonic behavior, confirming the presence of free charge carriers. At higher energies (>7 eV), *ε*_1_(*ω*) approaches zero, indicating diminished polarization at high-frequency excitations. The imaginary part, *ε*_2_(*ω*), directly corresponds to optical absorption arising from interband electronic transitions shown in Fig. 7(b). A pronounced peak appears at low photon energies for all compounds, which is attributed to transitions from occupied Sb-p and U-5f states to unoccupied transition-metal d states. As energy increases, *ε*_2_(*ω*) gradually decreases, indicating reduced optical transitions at higher photon energies. The similarity in spectral profiles among U_3_TiSb_5_, U_3_VSb_5_, U_3_CrSb_5_, and U_3_MnSb_5_ reflects their comparable electronic structures, with minor variations caused by different transition-metal contributions. The absorption coefficient increases sharply from zero photon energy, confirming that all compounds exhibit strong absorption in the visible and ultraviolet regions shown in Fig. 7(c). High absorption values (∼10^6^ m^−1^ order) indicate efficient photon–electron interaction. Broad absorption maxima observed between approximately 3–6 eV arise from intense interband transitions involving U-5f, T-d, and Sb-p states. The sustained high absorption over a wide energy range highlights the potential of these compounds for UV optoelectronic and radiation-shielding applications. The energy loss function *L*(*ω*) describes the energy dissipated by fast electrons traversing the material and is closely related to plasma oscillations shown in Fig. 7(d). A prominent peak is observed in the higher energy region (∼8–10 eV), corresponding to the bulk plasmon resonance, where *ε*_1_(*ω*) crosses zero and *ε*_2_(*ω*) is minimal. The presence of strong plasmon peaks confirms the metallic nature of U_3_TSb_5_ compounds and suggests possible applications in plasmonic and energy-loss spectroscopy-based devices. The optical conductivity *σ*(*ω*), spectra show significant values starting from zero photon energy, which further supports the metallic or highly conductive behavior of all compounds shown in Fig. 7(e). A pronounced peak around 4–6 eV is associated with enhanced interband electronic transitions.

At higher energies, *σ*(*ω*) gradually decreases due to reduced transition probability. Among the studied compounds, slight differences in peak intensity reflect the role of transition-metal substitution in tuning electronic transport properties. The refractive index *n*(*ω*) exhibits large static values at low photon energies, indicating strong light–matter interaction shown in Fig. 7(f). As photon energy increases, *n*(*ω*) decreases monotonically and approaches unity at high energies, implying reduced refraction in the ultraviolet region. The high refractive index in the low-energy regime makes U_3_TSb_5_ compounds promising candidates for optical coatings and photonic devices, where strong refractive control is required.

### 3.7 Thermoelectric properties

**Fig. 8:**
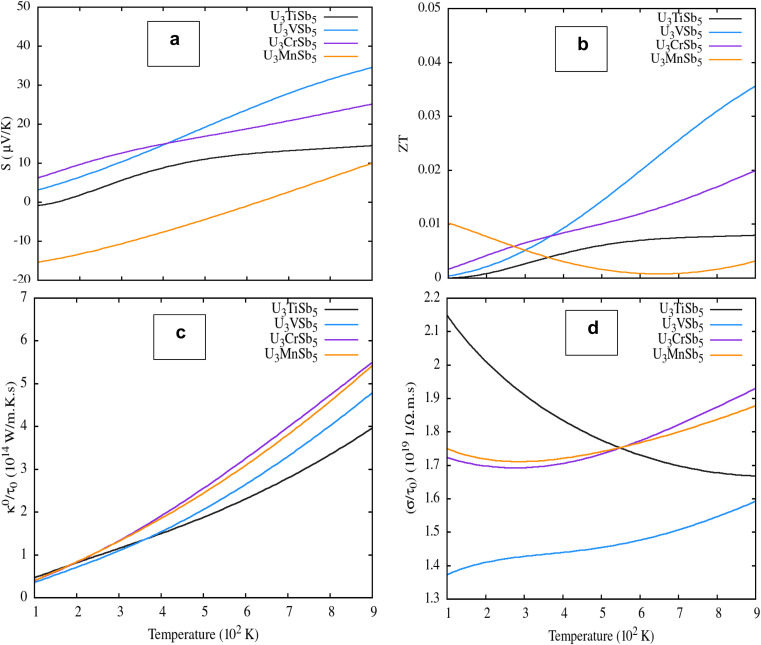
Temperature-dependent thermoelectric transport properties of U_3_TSb_5_ (T = Ti, V, Cr, Mn): (a) Seebeck coefficient (*S*), (b) dimensionless thermoelectric figure of merit (*ZT*), (c) thermal conductivity scaled as *κ*/*τ*, and (d) electrical conductivity scaled as *σ*/*τ* as a function of temperature.

Fig. 8 illustrates the temperature-dependent thermoelectric transport properties of U_3_TSb_5_ compounds across the 100–900 K temperature range. Regarding the Seebeck coefficient (panel a), U_3_VSb_5_ achieves the highest positive value (∼35 µV K^−1^ at 900 K), while U_3_MnSb_5_ displays negative values at low temperatures, suggesting electron-dominated transport, with all compounds showing increasing |S| trends with rising temperature. The dimensionless figure of merit *ZT* (panel b) reveals U_3_VSb_5_ as the best-performing compound, reaching ∼0.032 at 900 K, whereas U_3_MnSb_5_ peaks early near ∼0.01 before declining, and U_3_TiSb_5_ alongside U_3_CrSb_5_ remain notably low throughout the entire range. For thermal conductivity (panel c), all compounds exhibit monotonically increasing κ/τ with temperature, with U_3_CrSb_5_ and U_3_MnSb_5_ converging at higher values (∼5.4 × 10^14^ W m^−1^ K^−1^ s^−1^), while U_3_TiSb_5_ maintains the lowest thermal conductivity, which is generally favorable for thermoelectric performance. The electrical conductivity (panel d) shows U_3_TiSb_5_ peaking near 500 K before declining, U_3_VSb_5_ decreasing steadily, and U_3_CrSb_5_ with U_3_MnSb_5_ rising gradually, reflecting metallic-like behavior. Overall, U_3_VSb_5_ stands out as the most promising thermoelectric candidate, combining a high Seebeck coefficient, elevated *ZT*, and reasonable transport properties at high temperatures.

## 4 Conclusion

In this work, a comprehensive first-principles investigation of the actinide antimonide compounds U_3_TSb_5_ [T = Ti, V, Cr, Mn] has been carried out using density functional theory. The study systematically explored the structural, electronic, magnetic, mechanical, optical, thermoelectric and Phonon properties to assess their stability and multifunctional potential. Structural optimization confirms that all U_3_TSb_5_ compounds crystallize in the hexagonal *P*6_3_/*mcm* space group and are thermodynamically stable. The smooth energy–volume curves and well-defined equilibrium parameters obtained from Birch–Murnaghan fitting demonstrate robust structural integrity across the series. Transition-metal substitution at the T site induces moderate variations in lattice parameters and bulk modulus without altering the overall crystal symmetry, indicating good structural tolerance and compositional flexibility. Electronic structure calculations reveal that all compounds exhibit metallic behavior in both spin-up and spin-down channels, with multiple bands crossing the Fermi level. The density of states analysis shows that the electronic states near the Fermi energy are dominated by U-5f and transition-metal d orbitals, with Sb-p states contributing mainly at lower energies. Strong hybridization among these orbitals governs the metallic conductivity and spin-dependent electronic characteristics. Magnetic analysis establishes a ferromagnetic ground state for all investigated compounds. The total magnetic moments are primarily governed by uranium 5f electrons and transition-metal d electrons, with U_3_MnSb_5_ and U_3_CrSb_5_ exhibiting the largest magnetic moments. The presence of significant spin polarization together with metallic conductivity highlights the potential of these materials for spintronic and magneto-electronic applications. Mechanical property calculations confirm that all elastic constants satisfy the mechanical stability criteria for hexagonal systems. The evaluated bulk, shear, and Young's moduli indicate mechanically robust materials, while Pugh's ratio and Poisson's coefficient classify all compounds as ductile with mixed ionic–metallic bonding characteristics. The relatively low elastic anisotropy and moderate Debye temperatures further suggest good structural reliability and thermal stability. The optical response analysis demonstrates strong optical activity over a wide photon energy range. High dielectric constants at low energies, pronounced absorption in the visible-ultraviolet region, well-defined plasmon peaks in the energy loss spectra, and high optical conductivity confirm the metallic nature and highlight the suitability of U_3_TSb_5_ compounds for optoelectronic and photonic applications. Thermoelectric transport calculations show that the Seebeck coefficient and figure of merit (*ZT*) increase with temperature for all compounds. Although the *ZT* values are moderate, the combination of reasonable Seebeck coefficients, high electrical conductivity, and tunable thermal transport suggests that these materials could be further optimized for thermoelectric applications through doping, strain engineering, or nanostructuring. This study establishes U_3_TSb_5_ [T = Cr, Mn, Ti, and V]as a promising class of metallic, ferromagnetic, mechanically stable, optically active, and thermoelectrically tunable materials. The phonon dispersion analysis indicates that all studied U_3_TSb_5_ [T = Ti, V, Cr, Mn] compounds exhibit dynamical instability due to the presence of imaginary phonon modes. The strong coupling between structure, electronic states, and transport properties provides valuable insight into actinide-based intermetallics and offers a solid theoretical foundation for future experimental validation and functional material design.

## Conflicts of interest

The authors declare no competing interests.

## Data Availability

All supporting data are contained within the manuscript.
